# Correlation between Body Mass Index (BMI) and Performance on the Montreal Cognitive Assessment (MoCA) in a Cohort of Adult Women in South Africa

**DOI:** 10.1155/2022/8994793

**Published:** 2022-02-02

**Authors:** Antonio G. Lentoor, Lezani Myburgh

**Affiliations:** Department of Clinical Psychology, School of Medicine, Sefako Makgatho Health Sciences University, Ga-Rankuwa, 0208 Gauteng, Pretoria, South Africa

## Abstract

**Objective:**

Recent evidence suggests that obesity is increasing worldwide and may negatively impact neurocognition. Local studies on the association of weight status with neurocognitive function are sparse. This study is aimed at examining the association between body mass index (BMI) and neurocognitive functioning scores in a cohort of adult women.

**Methods:**

A convenience sample of 175 women aged 18 to 59 years (28.03 ± 8.87) recruited in a community-based quantitative study completed the Montreal Cognitive Assessment (MoCA). The BMI metric was used to measure body fat based on weight and height and was stratified as high BMI (overweight or obese) or low BMI (normal weight). The Beck Depression Inventory (BDI) was used to assess depression. Pearson's correlation analysis and the student's *t*-test analysis were performed.

**Results:**

We observed a significant inverse association between BMI and performance on MoCA (*r*(173) = −0.32, *p* < 0.001). Performance on subtest of attention, memory, constructive abstraction, and executive functions significantly and inversely correlated with BMI. Significantly lower scores on the MoCA were found in women with a high BMI compared to women with a low BMI (23 ± 4 vs. 26 ± 3), *t*(173) = 4.12, *p* < 0.0001).

**Conclusions:**

BMI and MoCA were inversely associated on both global and domain-specific neurocognitive test of attention, memory, and executive function; key neurocognitive control; and regulatory functions underlying behavior and decision-making. The findings provide a rationale for further research into the long-term effects of BMI on neurocognition.

## 1. Introduction

In the past decades, weight problems increased, especially overweight and obesity have become well-known public health problems that impact more than a billion people worldwide (1). The National Institute of Health (NIH) uses body mass index or BMI to provide an estimate of body fat in individuals of any age. An individual is classified as overweight with a BMI higher than 25, whereas a body mass index greater than or equal to thirty kilograms (BMI ≥30 kg/m^2^) indicates obesity (2). The World Health Organization (WHO) recognized adiposity as a global epidemic that increases the risk of many chronic diseases, including hypertension, type 2 diabetes, metabolic syndrome, cardiovascular disease, and strokes (3). It is well established in existing literature that higher body mass index (BMI) is not only associated with increased risk of chronic diseases but also the risk of all-cause mortality (3, 4).

Furthermore, recent evidence has linked high body mass index and deficits in neurocognitive function (5, 6). Neurocognitive function encompasses a diverse range of cognitive processing, integration, storage, and retrieval of information that facilitates complex initiation, planning, and regulation of behavior, thinking, and emotions. Neurocognitive function includes learning, memory, emotional regulation, and executive functioning (7–11). Neurocognitive impairment is defined as difficulty in remembering, learning new things, concentrating, or making decisions that impacts daily functioning. It is a major concern because it results in functional disability and may adversely affect the management of patients with obesity. The Montreal Cognitive Assessment (MoCA) has been found to be a reliable neurocognitive screening assessment and has shown to correlate significantly with BMI (12, 13).

Evidence shows that individuals with a high BMI or obese individuals exhibit deficits in multiple neurocognitive domains, including attention, memory, and executive function (9, 14). The evidence regarding the association between BMI and neurocognitive function exists even after controlling for comorbid health conditions (15). Notably, the findings in adults aged 19 to 65 years, similar to that of children (aged 4-18 years) which consistently shows that obesity being associated with poor neurocognitive performance (16). Furthermore, neurocognitive deficits are most consistently found in the executive function domain (17).

Noteworthy, in the elderly, however, inconsistent associations have been found between BMI and neurocognitive function (11, 18). Some studies found a negative association; others found a positive association, while some studies found no associations (19–21). This has been shown in studies conducted in both western (22) and eastern countries such as China (17). Bryant and colleagues examined the relationship between body mass index (BMI) and neurocognitive function among older black African American, Hispanic, and non-Hispanic white individuals and found that a negative association was in the white group and only overweight black African American group, whereas a positive association was found in the Hispanic group. Sturman et al. (23) in a longitudinal study from 1993 to 2003 examined the neurocognitive function and found that a higher BMI was associated with less neurocognitive decline when adjusting for comorbid illness in an elderly sample.

The exact mechanism of obesity-related neurocognitive dysfunction is complex. Prior studies consistently showed that the pathophysiological mechanisms involving vascular changes, impaired insulin and glucose regulation, systemic and central inflammation, gut dysbiosis, oxidative stress, and endothelial dysfunction are some of the explanations of obesity-related cognitive impairment and are perhaps telling of the difference found in adult and older age populations (24).

Despite these aforementioned findings, BMI-related neurocognitive function remains unclear and limited in developing countries. The association of body mass index with cognition remains to be fully understood in a South African population. To do so, we examined the association between BMI and neurocognitive function in a cohort of South African women. Based on the existing literature, we expected that a higher body mass index (BMI) would be associated with poorer neurocognitive function based on low performance scores on the Montreal Cognitive Assessment (MoCA).

## 2. Material and Methods

### 2.1. Study Participants and Design

We conducted a cross-sectional quantitative study, using a convenient sample of 175 women aged 18-59 years that were recruited from either the local hospital, university, or community from January to December 2019. All the procedures were conducted in accordance with the Declaration of Helsinki (25), and the study protocol was approved by Sefako Makgatho University Research Ethics Committee (SMUREC/M/206/2018: PG). Signed written informed consent was obtained from all participants. The participants were advised the opportunity of participation in the study by advertisement and word of mouth. Participants who were age ≥ 60 years, nonfluency in English, and active neurological and psychiatric conditions that could influence cognitive performance or interfere with participation were excluded. All the participants were first screened to meet the inclusion criteria. Thereafter, they completed a self-reported Beck Depression Inventory and recorded any comorbid health conditions such as hypertension and type 2 diabetes. Furthermore, the participants completed an anthropometric and neurocognitive assessment, which was conducted by a trained registered student clinical psychologist and confirmed by an experienced licensed clinical psychologist.

### 2.2. Demographic and Clinical Variables

The race/ethnicity, age, employment status, schooling level, and medical information, including hypertension and type 2 diabetes, were recorded, while all participants completed the self-assessment Beck Depression Inventory-BDI scale as a measure of depression. The BDI is frequently employed as a measure of depression in studies of obesity with a high internal consistency (Cronbach alpha = 0.91) (26).

### 2.3. Anthropometric Assessment

Each participant's height was measured bare feet with measuring tape. Participant's weight was measured to the nearest kilogram (kg) with a digital stadiometer. Body mass index (BMI) was defined as body weight (kg) divided by square of height (m). BMI were categories into low BMI (BMI ≤ 24.9 kg/m^2^) and high BMI (BMI ≥ 25.0 kg/m^2^) (2).

### 2.4. Neurocognitive Assessment

The Montreal Cognitive Assessment (MoCA) was used to assess the neurocognitive function (27). The test measure global cognitive function based on several cognitive domains includes short-term memory, visuospatial skills, executive function, attention and concentration, language, and orientation for a total of 30 points. Thus, the scores of the MoCA range from 0 to 30, higher scores indicate better neurocognitive function, and a score equal to or less than 26 (MoCA ≤ 26) can be used to define mild cognitive impairment (28).

### 2.5. Statistical Analysis

All data were coded and analyzed using SAS 9.3 (SAS Institute Inc., Cary, NC, USA). The data are presented as means ± standard deviations (SD) for continuous variables and frequencies for categorical variables. Correlation analysis was performed using Pearson's *r*-correlation coefficient. The comparison of anthropometric parameters (BMI) and neurocognitive function (based on MoCA test scores) was performed using Student's independent *t*-test. Two-sided *p* values <0.05 were considered statistically significant.

## 3. Results

### 3.1. Demographics and Clinical Variables

A total of 175 women aged 18-59 (28.03 ± 8.87) years had a mean BMI of 29.63 ± 10.51 kg/m^2^ (Table 1). Of the total sample, 57.14% had a low BMI ≤ 24.9 kg/m^2^, while 42.86% had high BMI of ≥25.0 kg/m^2^ (overweight to obese group). The mean age for participants with a low BMI was 23.7 ± 4.08 years, whereas mean age for those with a high BMI was 33.7 ± 2.19 (*p* < 0.001). Those that reported health comorbidities (*n* = 14, 8%) had a significantly higher BMI (*p* < 0.001). Forty-one percent reported mild depressive symptoms as a psychological comorbidity (BDI ≥ 10).

### 3.2. Association between MoCA Scores and Body Mass Index (BMI)

The average MoCA test score was 25 (SD = 3), with scores ranging from 15 to 30. MoCA scores were significantly inversely associated with BMI (*r*(173) = −0.32, *p* < 0.001). BMI correlated negatively with the MoCA composite scores for subtests executive function (*r*(173) = −0.19, *p* < 0.05), attention (*r*(173) = −0.25, *p* < 0.001), language (*r*(173) = −0.23, *p* < 0.05), constructive abstraction (*r*(173) = −0.22, *p* < 0.05), and memory (*r*(173) = −0.16, *p* < 0.05). The general linear regression model includes age, living standard levels (LSM), depression (BDI), and health comorbidities. After controlling for confounders (health comorbidities, age, and living standard levels), the results from the model explained 24% variance in BMI. The MoCA domain for attention showed a significant negative association with BMI, *B* = −1.183, *t*(153) = −1.94, and *p* = .054. The results, thus, indicated that for every point increase in participants' attention score, their BMI is expected to decrease by 1.183. When controlling for covariates, no significant associations between MoCA, *B* = −0.024, *t*(160) = −012, *p* = .905 and BDI total score, *B* = −0.11, *t*(160) = −0.11, and *p* = 0.913 with BMI. Age was positively associated with BMI; for every one-year increase in age, BMI is expected to increase by a factor of 0.636, *p* < .001.

The independent *t-*test found that individuals with a higher BMI compared to those with a lower BMI had a significant lower performance scores on the MoCA test (23 ± 4 vs. 26 ± 3), *t*(173) = 4.12, *p* < 0.0001 (Table 2). There was a significant age effect, *t*(173) = 7.30, *p* < 0.001, and women with a higher BMI were also significantly older (34 ± 2) than those with a lower BMI (23 ± 4). The independent *t*-test revealed no significant differences in MoCA scores, *t*(153) = −0.06, *p* = 0.96, between those with depressive symptoms (41%) and no depressive symptoms (59%). Similarly, no significant difference in MoCA performance was associated with health comorbidities.

## 4. Discussion

The association between body mass index (BMI) and cognitive performance is still debatable. To the best of our knowledge, this is the first study to attempt to describe the association between MoCA performance and body mass index (BMI) in a South African sample of adult women. The findings from this study showed that scores on the MoCA were negatively associated with BMI, indicating that lower mean score on the MoCA neurocognitive test is associated with a higher BMI in adult women. Our finding is consistent with previous studies reporting that higher BMI is inversely related to global neurocognitive function (24, 29, 30). In a recent study, Ciudin et al. (29) found individuals with a higher BMI (over weight and obese) performed significantly poorer on the MoCA compared to their normal weight counterparts. Similarly, Cournot et al. (31) investigated the relationship between BMI and neurocognitive decline in 2,223 healthy workers aged 32 to 62 years in their 5-year follow-up study and showed that a higher BMI at baseline was correlated with a higher neurocognitive decline at follow-up. Gunstad et al. (32) explored the association between BMI and neurocognitive performance in a cross-section of 408 healthy persons aged 20-82 years and found that participants with high BMI > 25 (overweight and obese) had poorer neurocognitive function than normal weight participants.

By contrast, our findings in this study is inconsistent with some previous studies that failed to show a link between obesity and neurocognitive functioning (18, 30, 33). In a review of existing studies, Buie et al. (34) attributed the inconsistencies to variability in study designs, choice of obesity measures, age inclusion criteria, and test used to measure cognitive functioning. The current study differs in that the sample was limited to 59 years old, with the high BMI group being older, falling in mid-late adulthood, and hence younger than the typical onset age for Alzheimer's disease (35). Kim et al. (11) found that the protective relationship between high BMI and cognitive function was not significant when age was confined to 45-65 years but was more apparent in persons 65 years or older. One of the reasons stated in research is that older aged people with a high BMI score experience slower decline in cognitive function than middle-aged individuals with a high BMI score, whereas Dye et al. (5) showed that mid-life obesity to be a risk factor of accelerated cognitive aging and developing Alzheimer's disease and vascular dementia in late life. Furthermore, in the majority of prospective studies, BMI has been utilized as the sole indication of adiposity. A number of factors and indices may influence BMI, which this study did not account for, including waist circumference and waist-to-hip ratio (WHR), both of which are more directly related to some unfavorable health consequences than BMI (35). Because muscle and fat are physiologically distinct and have different risk implications, using more sensitive measurements that differentiate components of body composition may be beneficial (36).

The results of this study also showed that obesity was negatively associated with performance on MoCA subdomain neurocognitive tasks of executive function, memory, and learning. This finding is consistent with previous studies (29, 37, 38). In fact, Benito-León et al. (37) in a study showed that participants who had higher BMI, overweight and obese, performed in the lowest quartile of global cognition and subdomains of executive function, memory, and learning. Likewise, other studies showed significant negative correlation between BMI and verbal memory, visual memory, and visual motor speed scores (9, 10). A review of previous studies (5) demonstrated that obesity was inversely correlated with subdomain of neurocognitive function, particular cognitive tasks involving decision-making, selective attention, memory, impulse, and executive control.

Several interrelated mechanisms may explain the obesity-cognitive dysfunction link (34, 39). Evidence from literature suggests that altered brain structure and neural activity, neural metabolic abnormality, oxidative stress, etc. mediate obesity's influence on cognition (34). It is possible that these mechanisms can help explain the participants' performance on the MoCA in this study. For instance, prior research showed that high BMI is associated with lower metabolic activity in areas of the prefrontal cortex, cingulate gyrus, and deficient white matter integrity in the uncinated fasciculus (40). These structures are particular important to executive functioning, learning, and memory, which was found to be associated with BMI in the current study. Deficits in this neurocognitive process underly poor decision-making and impulse control, lack of goal-directed behavior, and impaired independent function of individuals with obesity (41). While the focus of this study was not on the role of specific obesity-related mechanisms and underlying pathophysiological process, it does provide support of the relationship between BMI and neurocognitive (dys) function based on the MoCA scores. It perhaps reinforces the need for continued future prospective studies that will investigate the obesity-related neurocognitive dysfunction mechanisms, incorporating multiple adiposity indices and comprehensive objective neurocognitive assessments, to enhance the understanding of the findings from this study.

Lastly, we found no association between health comorbidity (i.e., type 2 diabetes and/or hypertension) and neurocognitive test scores. This finding is similar with the conclusions of Feinkohl et al. (42), who found that older persons who are obese had a greater prevalence of cognitive impairment regardless of comorbidities such as hypertension or diabetes. In contrast to the current finding, the ENBIND research found that type 2 diabetes in midlife (mean age 52 ± 8 years) women was associated with a minor but substantial cognitive decline (43). Likewise, we did not observe an association between psychological comorbidity (i.e., depression) and neurocognitive status. This result is congruent with the conclusions of a research conducted in Indonesia (44). Prior study found a relationship between comorbidities and lower neurocognitive test scores exclusively in older aged persons with greater BMI (45). The current research's age restriction may explain the discrepancy in findings, as the mean age for women with high BMI in this study was 33.7 ± 2.19 years. Unlike in the study by Yerrapragada and colleagues (46), we did not collect information on history and duration of comorbidities, current treatment, nonadherence, diet, and exercise. In previous studies, type 2 diabetes is known to be associated with decrements in memory and executive skills and information-processing if detected at the early stage of the disease (47). Importantly, the disparity in our study may be due to self-reported history, and the fact that we did not corroborate the self-reported medical history with medical records may have contributed to exposure bias (44).

This study has several potential limitations. Hence, the findings in this study require careful interpretation. First, because this was a cross-sectional design, no causal inferences can be drawn from the observed relationships in this study. Sampling bias is inherent in this study since nonprobability sampling was used to recruit the community-based sample of black African women. Another limitation is the use of only BMI and no additional adiposity measures. BMI does not discriminate between body fat mass and lean mass. Further distinguishing the significance of body composition using more sensitive metrics may be significant, given that muscle fat and lean mass are metabolically different and have different risk implications. Research employing BMI as a variable has limitations, as indicated by the inconsistent findings in previous studies. Further studies that would adjust for confounding factors: fat free mass, lifestyle, sociodemographic, diet, and other factors are needed. Lastly, it is important to consider that the MoCA is a cognitive screening tool, and while widely used in clinical settings in low-middle income countries, it has not been normed for the population. The findings of this study have important public health implications, particularly for interventions to support the retention of normal cognitive function in view of rising life expectancy globally. It can be useful for preventative interventions, including physical, dietary/nutritional, and lifestyle modification counseling to lower the cognitive risk linked with obesity. Improving cognitive capability and providing some protection against later-life cognitive decline and the risks of neurodegenerative disorders such as dementia later in life are imperative.

## 5. Conclusions

The results of this study provide evidence of an inverse association between BMI and MoCA test performance, both on global and domain-specific neurocognitive tasks of attention, memory, and executive function; key neurocognitive control; and regulation functions that underlie behavior and decision-making. The mechanisms underlying this inverse association is unclear at present. This finding provides a good rational to investigate the longitudinal effects of multiple indices of body composition on cognition with reliable objective neuropsychological tests battery in a large multicenter, prospective cohort design to understand the underlying mechanism.

## Figures and Tables

**Table 1:** Demographic and clinical characteristics of all the participants in the study.

Characteristics	All participants (*n* = 175)
Female (%)	100
Age (years)	
Range	20-54
Mean ± SD	28.02 ± 8.86
Highest level of education (%)	
High school	47.4
Diploma	8
Bachelor's degree	37.1
Postgraduate degree	7.4
Occupation status (%)	
Employed	24.6
Self-employed	2.3
Unemployed	58.3
Other (student)	14.7
BMI (kg/m^2^)	
Range	18.5-71
Mean ± SD	29.63 ± 10.51
Health comorbidity (%)	
Yes	8
No	92
Psychological comorbidity	
Mean BDI	9.77 ± 8.39
BDI score ≥ 10 (%)	40.6

Note: Values are presented as number (%) or mean ± standard deviation. Abbreviations: MoCA: Montreal Cognitive Assessment; BDI: Beck Depression Inventory; BMI: body mass index.

**Table 2:** MoCA and BMI characteristics of all the participants in the study.

Characteristics	Mean MoCA (SD)	95% confidence interval		
		Df	*t*	*p* value
Overall BMI (29.63 ± 10.52)	25 ± 3	1	4.12	0.0001^∗^
BMI ≤ 24.9 kg/m^2^ (22.27 ± 1.91)	26 ± 3			
BMI ≥ 25.0 kg/m^2^ (39.46 ± 9.17)	23 ± 4			

Note: Values are presented as number (%) or mean ± standard deviation. *t*: independent sample *t*-test. ^∗^*p* values were calculated and indicated significant association when values were <0.05.

## Data Availability

The raw data supporting the conclusions of this article will be made available by the authors, without undue reservation.
